# Retropharyngeal calcific tendinitis in the neurological emergency unit, report of three cases and review of the literature

**DOI:** 10.2478/raon-2023-0045

**Published:** 2023-11-30

**Authors:** Tatjana Filipovic, Jernej Avsenik

**Affiliations:** Institute of Clinical Neurophysiology, Division of Neurology, University Medical Centre, Ljubljana, Slovenia; Institute of Radiology, University Medical Centre, Ljubljana, Slovenia

**Keywords:** retropharyngeal calcific tendinitis

## Abstract

**Background:**

Retropharyngeal calcific tendinitis (RCT) is a relatively benign condition of calcination of the longus colli muscle tendon of unknown origin, which causes severe acute neck pain. However, it is often not recognised, which leads to delayed diagnosis and unnecessary treatment.

**Patients and methods:**

We have searched PubMed and Google Scholar for publications which reported at least one patient with RCT and were published in the last 20 years. The literature was then analysed according to the PRISMA-S protocol. We also report three patients with RCT presenting at the Neurological Emergency Unit, University Medical Centre, Ljubljana, Slovenia, from 1 January 2020 to 1 June 2022. We discuss their clinical presentation and differential diagnosis, explain our decision-making process, and briefly describe the clinical course. Case reports have been performed according to the CARE protocol.

**Results:**

We have analysed a total of 112 titles with 231 patients. The most frequent symptoms and signs were: neck pain, neck stiffness and odynophagia, as was the case in our reported cases.

**Conclusions:**

RCT is a dramatic yet self-limiting condition, often not recognised in time. An effort should be made to increase neurologists’ awareness about this condition.

## Introduction

Calcifications in the retropharyngeal space as a cause of severe acute head and neck pain have long been recognised, but have only recently been incorporated in the latest (3^rd^) International Classification of Headache Disorders (ICHD-3). Retropharyngeal calcific tendinitis (RCT) is, according to ICHD-3 criteria, a “headache caused by inflammation or calcification in the retropharyngeal soft tissues”.^1^ It occurs as a result of poorly understood mechanisms in the upper fibres of the longus colli muscle. Data from a similar condition affecting the shoulder joint showed neovascularisation and new nerve growth as a result of the innate immune response.^2^ This often dramatic but self-limiting condition is often not recognised among physicians, which leads to unnecessary diagnostic procedures and delayed treatment.^3,4^ Besides neurologists, other specialists are also involved in treatment of this condition, especially otorhinolaryngologists (ENT)^3,4^ and orthopaedic surgeons.^5^ The annual incidence of RCT is estimated to be from 0.5 cases per 100 000, up to 1.1 case per 1000.^4,6^

The aim of the present study was to review the literature data and present our own experience with RCT in order to increase neurologists’ awareness about the condition.

## Patients and methods

On 7 September 2022 we have searched the PubMed and Google Scholar with the keywords “retropharyngeal calcific tendinitis”, “longus colli tendinitis” and “acute neck pain”. The search was conducted for the titles published in the last 20 years (2002–2022). The analysis of the literature was performed according to the PRISMA-S protocol.^7^ We analysed the frequency of reports of RCT regarding the facility where patients were first registered and treated.

In addition, we report three cases of retropharyngeal calcific tendinitis (RCT) that were referred to the Neurological Emergency Unit (NEU), Division of Neurology, University Medical Centre Ljubljana, Slovenia, from 1 January 2020 to 1 June 2022. The reports follow the CARE protocol.^8^

### Patients

NEU is a tertiary medical facility for over 300 000 inhabitants where 25 016 patients were examined in the time period described above, of which 2073 were discharged with the main diagnosis “Headache, unspecified” (G 44.8 or R 51) according to ICD-10 and only three were diagnosed with RCT (M 65.2).

#### Patient 1

**FIGURE 1.**
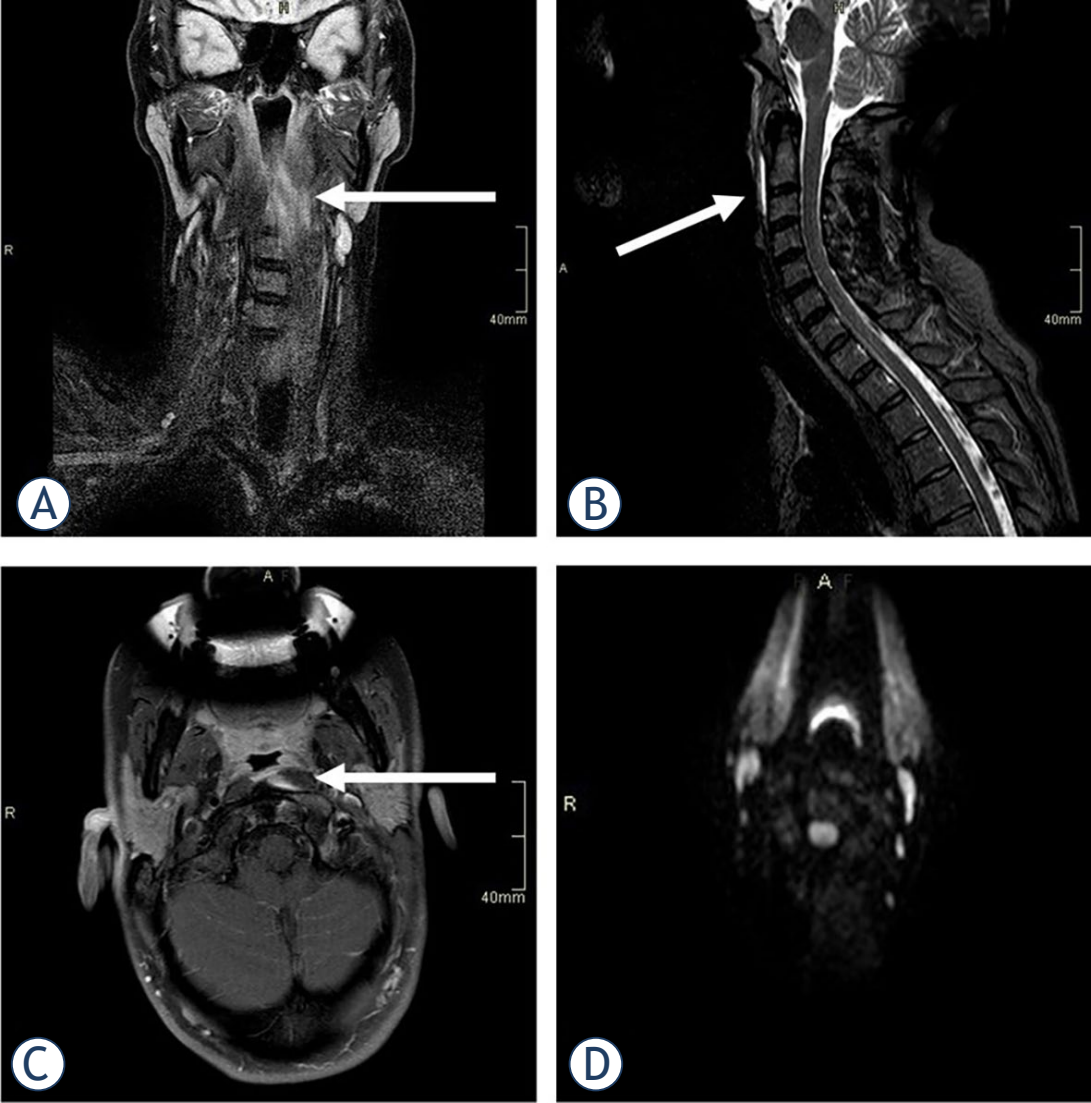
MRI in a 40-year-old female showed short tau inversion recovery sequence (STIR) hyperintensity in the upper part of her left Longus colli muscle, suggesting an oedema **(A)**, with thin prevertebral effusion on sagittal images **(B)**. After intravenous gadolinium contrast injection, a small area of enhancement was observed in the medial aspect of the muscle **(C)**, but no peripherally enhancing collection to suggest an abscess was present. Diffusion-weighted imaging was normal, excluding the presence of pus **(D)**.

A previously healthy 40-year-old female with suspected meningitis was referred to our institution in November 2020. She was experiencing excruciating throbbing neck pain, which had developed spontaneously within 12 hours without any trauma or heavy mechanical load. She complained that swallowing was painful and that the pain increased with any attempt to move the head. A neurological exam showed severe neck stiffness with immobility in all directions as well as dysesthesia over the vertex and occipital regions. Laboratory workup revealed only mildly increased C reactive protein (CRP) of 20 mg/l (normal value < 5 mg/l) and white blood cell count (WBC) of 10.2 (normal < 10×10^9^/l). Although the patient was afebrile, the retropharyngeal abscess was still considered in the differential diagnosis. Magnetic resonance imaging (MRI) of the neck showed fluid collection and swelling in the cranial part of the longus capitis/colli muscle on the left (Figure 1). A lumbar puncture was also performed, but the CSF was normal. She was treated in the NEU and discharged with ketoprofen 200 mg daily and a soft neck collar. The pain resolved in one month.

#### Patient 2

A 51-year-old female with a history of arterial hypertension was referred to the NEU in November 2021 with clinical suspicion of meningitis. Five days prior to referral, she experienced a sudden mild pain in the right posterior neck, radiating to the occipital region. In the following three days, the pain became unbearable. It increased during swallowing and with eye movements. Due to the chronic neck pain, the patient had an MRI of the neck 9 months prior to the examination. Broad-based protrusions of the C5/6 and C6/7 intervertebral discs without compromise of neural structures were described, but no other abnormalities were noted at the time. She was afebrile at presentation. A neurological exam showed limited mobility of the neck and reduced light touch sense over the right lower extremity.

**FIGURE 2.**
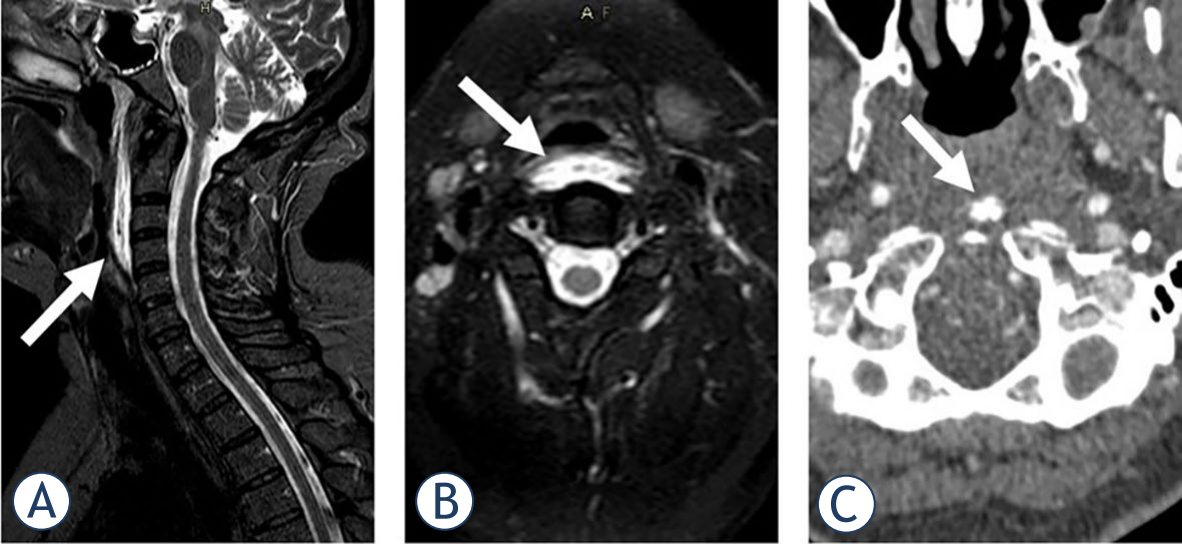
STIR (short tau inversion recovery) imaging in sagittal **(A)** and axial **(B)** plane demonstrated prevertebral soft tissue swelling and oedema in a 51-year-old female, suggesting retropharyngeal calcific tendinitis as the underlying cause. Calcifications in the medial aspect of the longus colli muscle in front of the C1 arc were noted on computed tomography angiography (CTA) **(C)**, confirming the diagnosis.

Laboratory workup was normal. Head CT and computed tomography angiography (CTA) of the aortocervical and intracranial vessels revealed no vascular abnormalities. MRI of the cervical spine revealed prevertebral oedema from the C1 to the C4 level (Figure 2), not seen on previous MRIs. In addition, calcifications in front of the C1 arc on CTA was noted. The patient had been treated three days in the hospital and discharged with ibuprofen, 1800 mg daily. The pain gradually subsided over the next two months. The sensory disturbance over the left leg remained unexplained.

#### Patient 3

In May 2022, a previously healthy 43-year-old female experienced pain in the right posterior neck which evolved gradually over a period of 48 hours. She described an electric shock-like pain that was radiating to the occipital area at any attempt of head extension, and even if she tried to hold the head in the neutral position. Laboratory workup at the Medical Emergency Unit revealed only slightly elevated CRP. Pain medication (metamizole 2.5 g IV) was minimally effective. Three days after the onset of pain the patient was examined at the NEU where occipital neuralgia was suspected, and outpatient MRI of the head and neck was suggested. As the pain continued, she came to the NEU on day 5 and spondylodiscitis was added to differential diagnosis. A neck CT revealed calcifications in the region of alar ligaments and repeated laboratory results showed elevated CRP of 20 mg/l (normal < 5 mg/l), with no other abnormalities. The patient was discharged with ibuprofen 1200 mg daily. As the pain continued without relief, she returned to the NEU on day 7. This time RCT was diagnosed and a short course of corticosteroids was prescribed (dexa-methasone, 4 mg per os daily). The pain disappeared within one week.

## Results

**FIGURE 3.**
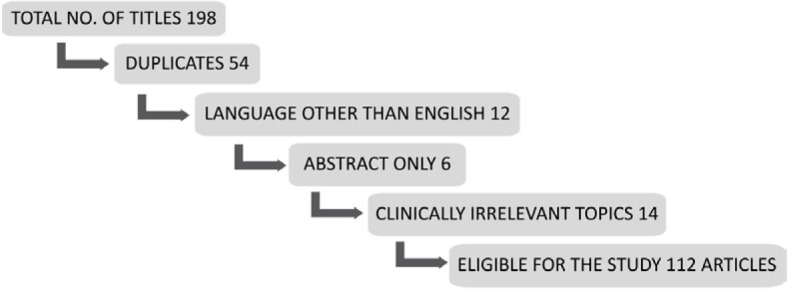
Flowchart of article selection.

The literature search returned no randomised controlled trials, meta analyses, clinical trials or systematic reviews. We retrieved a total of 198 titles (Online Resources- 1 and 2), all of which were case and series reports and reviews. Of those, 112 were eligible for the study. The details of article selection are given in Figure 3. The total number of reported cases was 231. The results of literature analysis are summarised in Table 1.

**TABLE 1.** Results from literature analysis

	**N**	**%**
**SPECIALITY REPORTS**	112	100
**Otorhinolaryngology (ENT)**	32	28.6
**Emergency medicine**	26	23.2
**Orthopaedic surgery**	24	21.4
**Other**	19	17
**Neurology**	11	9.8
**PATIENTS TOTAL**	231	100
**Sex: women:men**	121:110	52.4:47.6
Age (years)	22–78	46.7
**Median**		
**No comorbidities**	224	96
**Acute onset (24–72 hours)**	208	91
**LEADING SYMPTOMS**		
**Neck pain**	231	100
**Neck immobility**	222	96
**Odynophagia**	210	91
**Trismus**	35	15
**Torticollis**	11	5
**Stridor**	1	0.4
**Dysarthria**	1	0.4
**Vertigo**	1	0.4
**DIAGNOSTIC WORKUP**		
**Mild to moderate increase in CRP and/or total leucocyte count**	216	93
**CT**	111	43
**CT + MR**	120	47
**Aspiration biopsy**	7	3
**DIFFERENTIAL DIAGNOSIS**		
**Retropharyngeal abscess**	134	58
**Spondylodiscitis**	28	12
**Meningitis**	25	11
**Neck artery dissection**	4	1,7
**COURSE**		
**Marked improvement within 2 weeks**	221	95

## Discussion

According to ICHD-3 criteria, RCT represents a “headache caused by inflammation or calcification in the retropharyngeal soft tissues”. The pain is usually severe, continuous, throbbing or electrising in quality. Trigeminal afferent fibres from dura and cervical afferent fibres from the skin and muscular tissue in the cervical region converge to synapse onto the same second-order neurons in the trigeminocervical complex.^9^ This at least partially explains occipital (headache) and pharyngeal (odynophagia) irradiation of the pain as well as neck stiffness (or decreased range of motion), as seen in our patients. Torticollis is sometimes reported, but may represent exaggerated neck stiffness. A detailed review of the (rare) causes of craniofacial and neck pain can be found in the literature.^10^

Little is known about the causes of calcium deposition or inflammation in the longus colli muscle. One report on histological findings in the retropharyngeal tissue of RCT patients has revealed a foreign-body type of inflammation around hydroxyapatite crystals.^11^ Immunological mechanisms involving the innate immune system in the form of new nerve growth and neovascularisation within the tendon in calcific tendinitis of the shoulder joint have been reported.^12^ Similar processes in the retropharyngeal space may be a plausible explanation in patients with RCT. However, further studies are needed to confirm these speculations.

Differential diagnosis includes other similar conditions that should be promptly recognised by neurologists (Table 2).

**TABLE 2.** Differential diagnosis of the Retropharyngeal calcific tendinitis (RCT)

**Feature**	**RCT**	**Meningitis**	**Abscess**	**Discitis**	**Dissection**	**GON,CH**
**Neck pain**	+++	++	+++	+++	++	++
**Fever**	−	+	+	+	−	−
**Photophobia**	−	+	−	−	−	−
**Nausea**	−	+	−	−	−/+	−
**Decreased ROM**	+++	+ (flexion)	++	++	−	−/+
**Odynophagia**	++/+	−	++	−/+	−	−
**Long tract signs**	−	−	−	−/+	+	−

CH = cervicogenic headache; GON = greater occipital nerve neuralgia; ROM = range of movement

Meningitis and meningoencephalitis are usually accompanied by fever, photophobia, nausea and/or vomiting, or even an altered mental state^1^, not typically seen in RCT. They are also accompanied by neck stiffness, but only on flexion, not on extension or rotational movements.^1^

Odynophagia is often seen in RCT, but is a hallmark of retropharyngeal abscess, where in most cases, the blood count shows elevated leucocytes above 12 × 10^9^/l.^3,13^ Spondylodiscitis, another possible cause of acute severe neck pain, is usually accompanied by clinical and laboratory signs of systemic inflammation, and diagnosis is made after appropriate imaging.^14,15^ Spontaneous carotid or vertebral artery dissection could present as an isolated head and/or neck pain, sometimes accompanied by nausea, but no neck stiffness has been reported.^16^ Lastly, greater occipital nerve (GON) neuralgia and cervicogenic headache (CH), which are relatively benign conditions, characterised by prominent neck pain, should also be kept in mind1.^1,17^ However, pain in GON neuralgia emerges in the form of short (seconds to minutes) attacks, leaving tenderness or allodynia in the region of GON.^1^ Therefore, when pain evolves over a period of several hours or even days, other causes should be considered. CH is a broad category of chronic conditions, with some diagnostic criteria overlapping with RCT.

Radiologically, differential possibilities in patients with suspected RCT include retropharyngeal abscess, tumour or even trauma; therefore, familiarity with typical imaging findings facilitates early diagnosis and may prevent inappropriate therapeutic procedures (Table 3).

**TABLE 3.** Radiological clues for differential diagnosis

	**Differential diagnosis**		

**Modality**	**RCT**	**ABSCESS**	**TUMOUR**
**X-RAY**	May show calcifications	Prevertebral swelling	− Prevertebral swelling
**CT**	Calcifications LCM oedema	+Peripheral enhancement +Lymphadenopathy	+Soft tissue mass (Variable enhancement) +Lymphadenopathy
**MR**	May suggest calcifications LCM oedema	+Diffusion restriction (pus)	(Superior contrast resolution)

LCM = longus colli muscle

As plain film radiography may miss subtle calcifications within the tendon, CT is the preferred imaging modality due to its supreme resolution and multiplanar capabilities.^18^ CT reliably confirms the location of calcifications within the superior fibres of the longus colli tendons and may show soft tissue swelling and/or small retropharyngeal effusion as well.^19^ Nowadays, the first imaging modality that these patients undergo is usually MRI, which demonstrates oedema and retropharyngeal effusion clearly. However, as calcifications are much subtler on MRI in comparison to CT, a high level of clinical suspicion is needed for the correct diagnosis. On the other hand, the presence of peripheral postcontrast enhancement or the evidence of pus on diffusion-weighted imaging should suggest infection as the cause.^14^ CT is immediately available and cheap but exposes the patient to ionizing radiation, which may be inappropriate in women in the childbearing age. MRI is not as accessible as CT, is expensive, and lasts longer, which may pose a problem due to inability of the patient to lay still in the supine position. Finally, MRI may be contraindicated in the cases of metallic implants or claustrophobia.

## Conclusions

RCT is a rare disorder that neurologists should be familiar with. It is also a relatively new subject for neurologists: it has been recognised as a neurologic disorder only in 2013, when the ICHD-3 criteria were published. Typically, the patient is a previously healthy middle-aged person, presenting with the triad of neck pain, neck immobility and painful swallowing. Mildly or moderately elevated CRP and leucocytes are usually present. Neck MRI and/or CT imaging should be performed to confirm diagnosis. Treatment with oral steroids or non-steroidal anti-inflammatory drugs should lead to prompt symptom alleviation.
